# AI for PET image reconstruction

**DOI:** 10.1259/bjr.20230292

**Published:** 2023-09-04

**Authors:** Andrew J Reader, Bolin Pan

**Affiliations:** 1 School of Biomedical Engineering and Imaging Sciences, King’s College London, London, UK

## Abstract

Image reconstruction for positron emission tomography (PET) has been developed over many decades, with advances coming from improved modelling of the data statistics and improved modelling of the imaging physics. However, high noise and limited spatial resolution have remained issues in PET imaging, and state-of-the-art PET reconstruction has started to exploit other medical imaging modalities (such as MRI) to assist in noise reduction and enhancement of PET’s spatial resolution. Nonetheless, there is an ongoing drive towards not only improving image quality, but also reducing the injected radiation dose and reducing scanning times. While the arrival of new PET scanners (such as total body PET) is helping, there is always a need to improve reconstructed image quality due to the time and count limited imaging conditions. Artificial intelligence (AI) methods are now at the frontier of research for PET image reconstruction. While AI can learn the imaging physics as well as the noise in the data (when given sufficient examples), one of the most common uses of AI arises from exploiting databases of high-quality reference examples, to provide advanced noise compensation and resolution recovery. There are three main AI reconstruction approaches: (i) direct data-driven AI methods which rely on supervised learning from reference data, (ii) iterative (unrolled) methods which combine our physics and statistical models with AI learning from data, and (iii) methods which exploit AI with our known models, but crucially can offer benefits even in the absence of any example training data whatsoever. This article reviews these methods, considering opportunities and challenges of AI for PET reconstruction.

## Introduction

Conventional implementation of image reconstruction for positron emission tomography (PET) involves writing computer code, a series of programming steps called an algorithm, based on an understanding of the physics and statistics of PET imaging. Using this understanding, the measured data from a PET scanner (*e.g.* sinogram data,^1^ histoimages^2^ or list-mode data^3^) are processed by the algorithm to form a reconstructed image. The term “image” refers to a representation of the spatiotemporal distribution of the PET radiotracer within the subject in the scanner.^4^ Usually this is a two-dimensional (2D) or three-dimensional (3D) array of pixels or voxels with values that are estimates of the radiotracer’s concentration in each of those pixel or voxel regions of the scanner field of view (FOV).

Conventionally, imaging scientists assume they know a good physics model and a good statistical model for both the signal and the noise in the PET measured data. By solving a mathematical description of the measured data, they implement the solution by fixed computer code, to reconstruct an image based on these modelling assumptions. Example reconstruction methods include iterative expectation maximisation (EM) algorithms^5^ for maximum Poisson likelihood reconstruction,^6,7^ which use a model of the imaging physics (the system or forward model^8^) for the signal, and a Poisson model for the noise. Such reconstruction methods are based on human intelligence, understanding and mathematical problem solving. In stark contrast, image reconstruction that is based on artificial intelligence (AI), such as methods using supervised deep learning^9^ (*e.g.* AUTOMAP^10^ or DeepPET^11^), seeks instead to *learn* the sequence of steps of the computer code that is needed to reconstruct from measured data. This learning process, the training of an AI model, relies on many tens of thousands of examples, or samples, where each sample is composed of a PET measured dataset paired with the known or desired image output that is expected from that measured dataset. This approach learns the parameters of a deep network (effectively the programming steps of the computer code) by way of examples, rather than relying on an assumed model of the PET imaging process. Most research into AI for PET image reconstruction takes place in the middle ground between these two extreme cases, such that the overall algorithm neither relies purely on modelling assumptions based on human intelligence nor purely on AI learning from example data pairs. A further burgeoning area of AI research for PET reconstruction uses AI to learn how to reconstruct even from just a single measured dataset in hand, without reference to any other external example data, as will be described later in this article. First though, it is useful to review the state-of-the-art conventional reconstruction methods, which assume that all models are known well via human intelligence.

### Conventional reconstruction: filtered backprojection (FBP)

A well-known image reconstruction algorithm is filtered backprojection (FBP). FBP first filters the sinogram data and then backprojects the filtered data to obtain an image.^12^ Early versions of FBP were implemented by a convolution of each projection view, followed by backprojection of each filtered projection view. As a side note, one of the very earliest uses of machine learning for emission tomographic reconstruction was based on learning the convolution kernel for the filtering process.^13^

Image reconstruction by FBP is a basic but robust method, being predictable due to its linearity, even though often producing noisy images. Noise in the reconstructed image is usually handled by a simple post-reconstruction smoothing, requiring just one adjustable parameter (the degree of smoothing) to yield easy to interpret images for radiologists, where the noise and resolution level of the images is usually visually self-evident. One of the reasons that FBP often produces noisy images is its lack of modelling of the varying noise levels in the data, when in fact the count-limited PET data do vary in statistical quality and can be well modelled by a Poisson distribution. Furthermore, FBP is limited by an overly simple model of the signal in the sinogram data—the data are modelled by the Radon or X-ray transform.^14^ Hence, before using FBP, the sinogram data need to be first precorrected for scattered events, random events, photon attenuation and detector normalisation.^15^ The subtractions arising from removing scatter and randoms, followed by the amplifications arising from attenuation correction, can be particularly problematic for increasing noise. As a result, the sinogram data have even greater variations in statistical quality, yet FBP still treats all the data as equally important, resulting in still noisier reconstructions. Statistical image reconstruction methods, reviewed next, avoid this inappropriate uniform weighting of the data.

### Iterative reconstruction: maximum likelihood–expectation maximisation (MLEM) and OSEM

In the 1980s–90s, superior iterative methods became available for PET image reconstruction, such as the expectation maximisation (EM) algorithm for maximum likelihood (ML) reconstruction. MLEM,^6,7^ and its accelerated version, ordered subsets EM (OSEM),^16^ use an improved model of the noise in the data (*i.e.* Poisson counting noise, rather than Gaussian) and perhaps more importantly, allow improved modelling of the signal in the data by using known imaging physics. Hence, rather than a simple Radon or X-ray transform model (as used by FBP, and hence requiring problematic data precorrections), MLEM and OSEM allow many physical effects, such as attenuation and normalisation, to be explicitly modelled and included within the system model of the image reconstruction algorithm.^8^ By avoiding precorrection of the data, the projection data counts remain raw and unweighted (so projection bins with low counts due to attenuation are left unamplified) and such integer (count) data are modelled well by a Poisson distribution. One example of improving a reconstruction algorithm’s model of the signal in the data is the use of “PSF modelling”,^17–20^ where the point spread function (PSF) is modelled during the reconstruction. By including a PSF model as part of the system matrix or forward model, spatial resolution can be improved in the reconstructed images found by MLEM+PSF or OSEM+PSF. In contrast, modelling of effects such as the PSF within FBP is not usually done (however, PSF modelling was implicit in earlier 3D backproject then filter (BPF) methods when using measured point source data).^21,22^

### Advanced regularised iterative reconstruction: maximum *a posteriori* (MAP) methods

**Figure 1.**
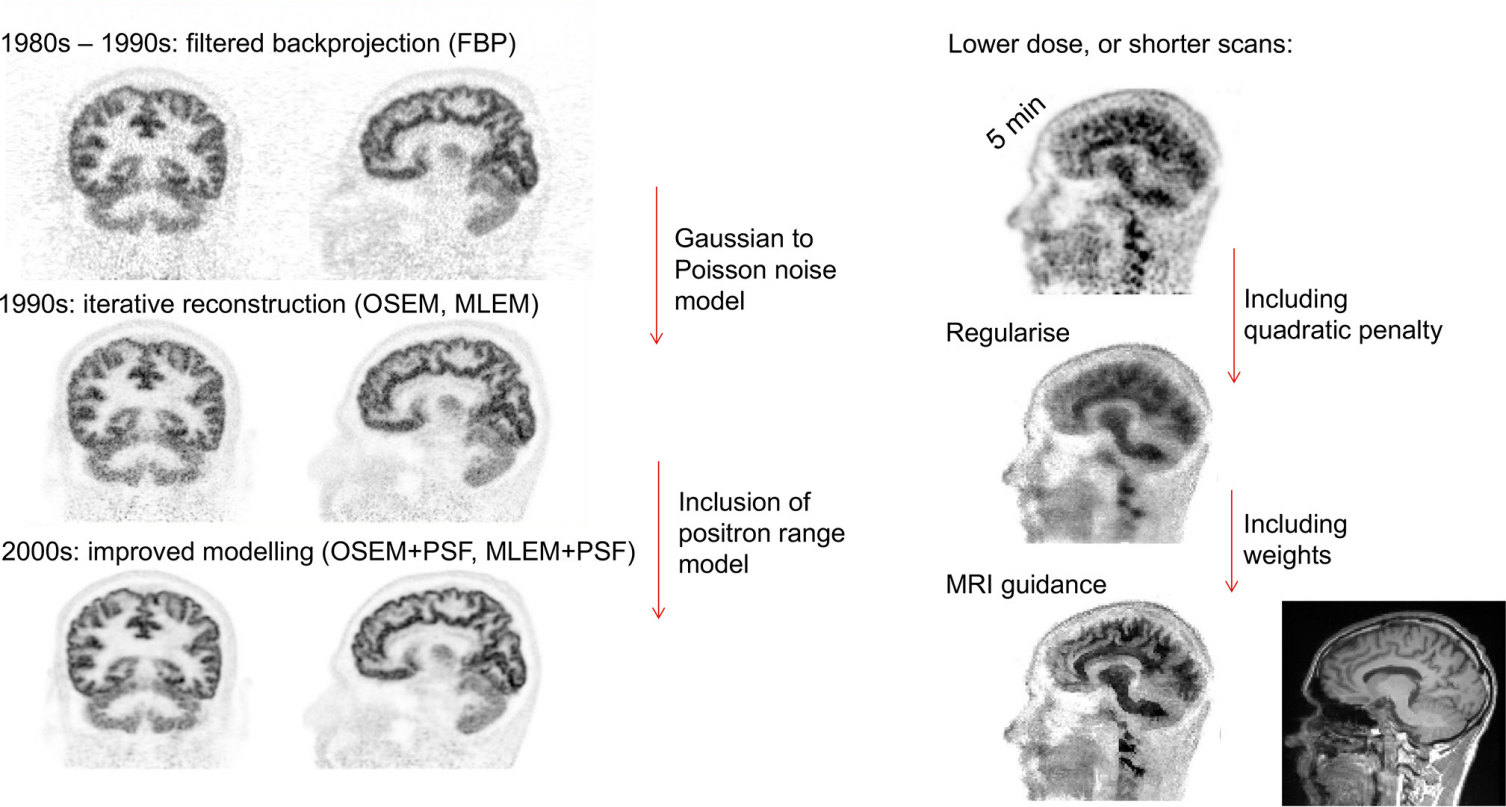
The progress of PET image reconstruction over the decades. On the left hand side, unregularised reconstruction methods started with 3D FBP, progressed into Poisson noise modelling with MLEM and OSEM, and then began to improve the model of the data with PSF modelling. On the right hand side (*shown for a different subject, tracer and scanner*), regularisation methods were adopted to compensate for noise, including anatomical guidance of the regularisation (*e.g.* via MRI). 3D, three-dimensional; FBP, filtered backprojection; MLEM, maximum likelihood–expectation maximisation; OSEM, ordered subsets expectation maximisation; PSF, point spread function.

Whilst MLEM and OSEM offer improved image quality for PET compared to FBP (as shown on the left side of Figure 1), there is nonetheless often a need to reduce scanning time and/or reduce the injected dose of the radiotracer. Consequently, noise remains an issue, and hence regularised (noise-compensated) image reconstruction methods are needed. These methods not only use: (i) an improved noise model (Poisson), and (ii) an improved model of the imaging physics, but they furthermore include (iii) a model of the type of images we expect to see, via a prior^23^ or penalty. A prior assigns a probability to any given candidate reconstructed image—assigning low probabilities to images we regard as improbable for the uptake of a PET radiotracer (*e.g.* erratically noisy images), and higher probabilities to images more likely to represent a tracer’s uptake (*e.g.* smoothly varying images). These regularised reconstruction methods consider both the likelihood of a candidate image given the measured data as well as the prior probability of any given image, and hence by seeking to maximise the posterior probability are called maximum *a posteriori* (MAP) methods (or, when using an EM algorithm, MAPEM methods).^24,25^ In many cases, the model of how we expect PET images to look takes the form of a penalty function rather than a probability, and such approaches should be called penalised likelihood (PL) methods. An example is Q-clear,^26^ which assumes that the relative differences between neighbouring pixel values in an image should not be too large (as large local differences are assumed to be associated with noise rather than with clinically useful image features).^27^ More recently, use of complementary structural information, such as from MRI, has been used to provide guidance to the regularisation,^28–31^ allowing preservation of large local differences in images that are due to legitimate image edges and features, rather than due to noise. Figure 1 summarises the progress in image reconstruction from PET, starting from the 1980s, following all the way through to methods developed over the >40 years which followed.

### Regularisation by change of spatial basis: kernel methods

**Figure 2.**
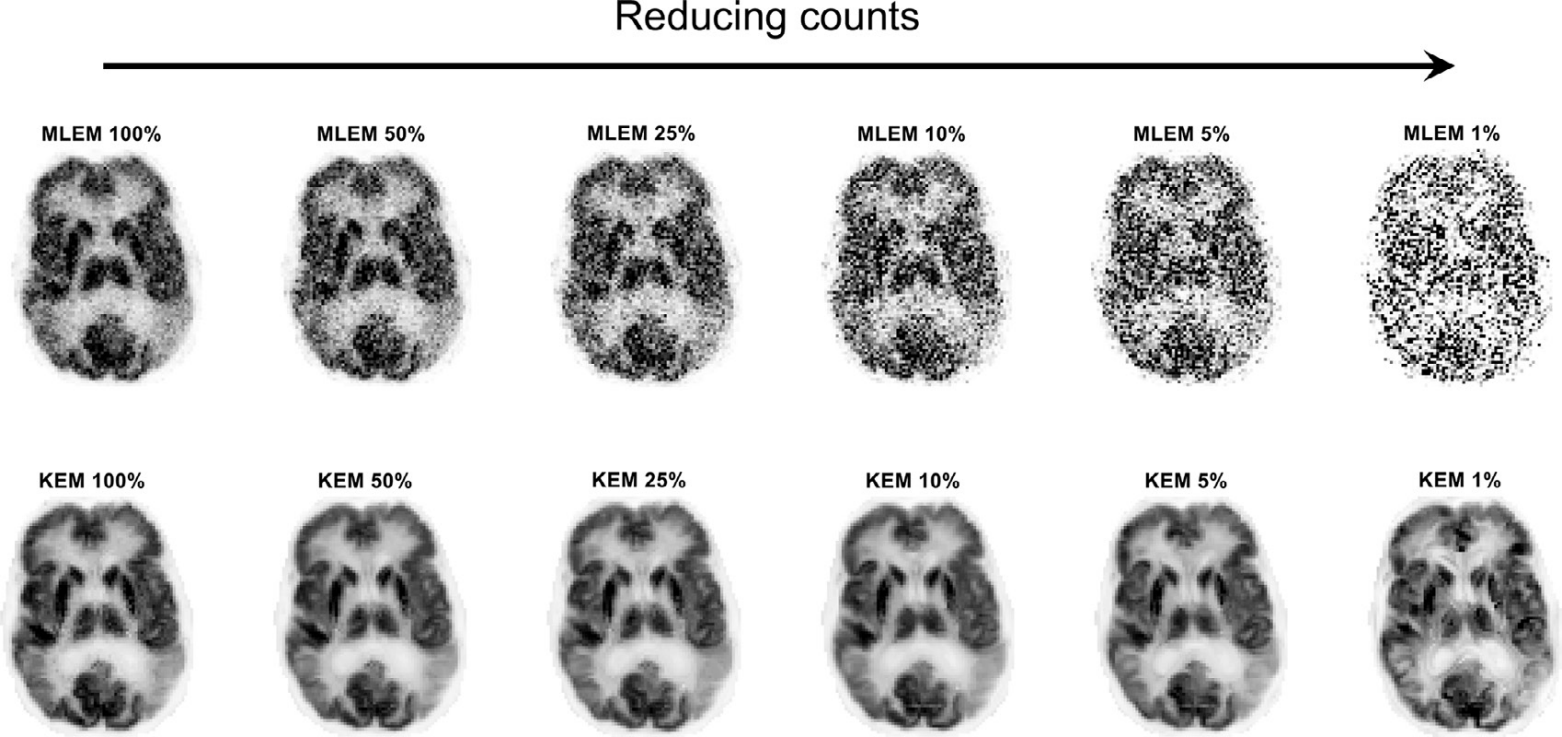
The kernel method for PET image reconstruction when using an MR image to provide the feature vectors for forming the spatial basis functions. This figure shows how this change of spatial basis can result in notable noise reduction even when just a small percentage of the original data are used.^34^ The original KEM^32^ was based on similarities of 3-point time–activity curves for each voxel when deriving the spatial basis functions. KEM, kernel method; MLEM, maximum likelihood–expectation maximisation; PET, positron emission tomography.

One further important category of conventional methods to mention is the kernel method (KEM), proposed by Wang and Qi in 2015.^32^ This method amounts to a change of spatial basis functions: instead of regarding an image as a collection of independent pixels or voxels with coefficients (amplitudes or values) being found for each one, the kernel method groups pixels according to their similarity in terms of some additional features. The feature data could simply be a 3-point time–activity curve (TAC) for each pixel, or could be the registered MR image value at each pixel, or even the patch of MR pixel values that are the neighbours of the pixel.^33^ By whatever means, each PET pixel has a list of additional values (a feature vector) associated with it. PET pixels can thus be grouped together into clusters, according to the similarity of these feature vectors. Conventional MLEM can then be used to find the coefficients for these pixel clusters (spatial basis functions), and the implicit regularisation achieved can be impressive: see Figure 2, where MR images were used to form the kernel spatial basis functions.^34^

## AI methods: from direct to iterative

Given the sophistication of conventional state-of-the-art image reconstruction methods, one might wonder whether further improvement is possible. However, conventional approaches suffer from simplified models and assumptions. While the models of the PET scanner (captured in the forward model or system matrix) may be of acceptable accuracy, and while the Poisson noise model may be appropriate, when it comes to modelling our expectations of the images, through a prior or a penalty, this is where conventional modelling is either too basic or too imposing. A simple noise compensation approach is to use a quadratic penalty,^35,36^ which amounts to neighbourhood smoothing of the reconstructed image values. This can be regarded as robust, but noise is reduced at the cost of spatial resolution, with edges in the images being compromised, or even small features or lesions being smoothed to become low contrast, even indiscernible, regions. If anatomical guidance information is used (*e.g.* from CT or MR images), this can be too imposing, as the user needs to decide how strongly to pay attention to the anatomical information. While anatomy can usefully guide the smoothing process of regularisation, it can be misleading, as there will invariably be structures and features in an MR or CT that are discrepant with those of the PET image for a given radiotracer.^30^ As a result, even spatial resolution and edge preserving guided regularisation can give poor results in some image regions,^36,37^ through smoothing away of some PET features while also wrongly enhancing edges or structures that are not truly present in the PET data. Hence, there is clearly scope for improving conventional reconstruction, whether by improving the physics or noise modelling, or, perhaps more significantly, by improving the modelling of the penalty or the prior (the probabilistic model of how we expect PET images to appear).

### Direct AI methods for PET reconstruction

**Figure 3.**
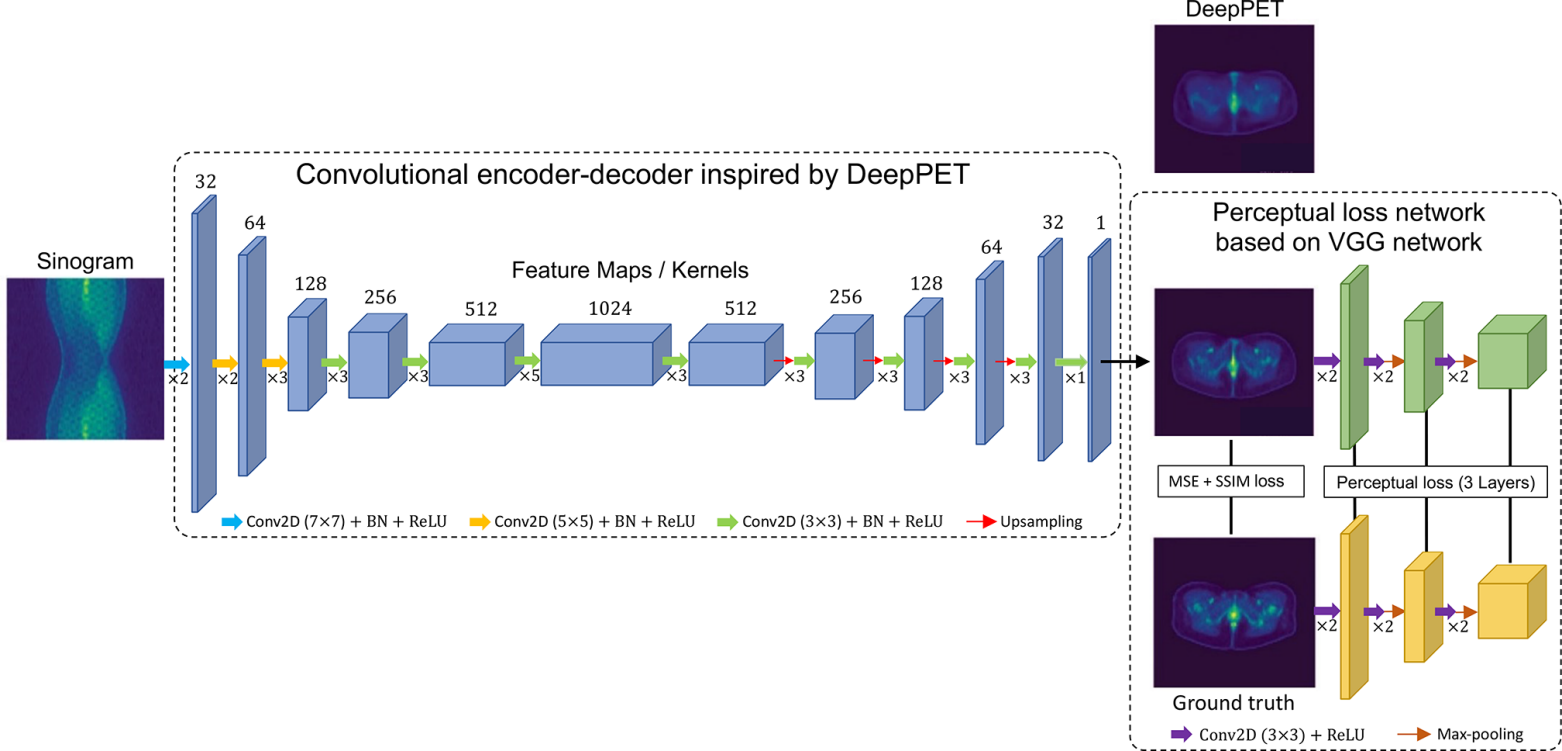
Representation of the performance of direct AI (purely data-driven) methods through the example of DeepPET^11^ and recent modifications.^39,40^ The example sinogram input and DeepPET reconstructed output are courtesy of Kuangyu Shi, related to their work on improving the performance for long-axial FOV PET, with their result shown as the output of the network here (image in row 2 of the figure).^40^ FOV, field of view; PET, positron emission tomography.

To start the review of AI for PET reconstruction,^38^ we will first consider the extreme case of ignoring nearly all modelling assumptions. Such approaches learn the entirety of the image reconstruction algorithm (*i.e.* the imaging physics, the data statistics and the prior model for the reconstructed images). Methods which seek to learn the whole mapping from measured data to a reconstructed image without use of known models will be referred to here as direct AI methods. A well-known example is DeepPET,^11^ shown in Figure 3, with example images shown along with a more recent adaptation^40^ of the approach for long-axial FOV PET imaging. The deep network starts with convolutional encoding layers, which learn to analyse the sinogram for small scale features at a given level of spatial resolution. The resulting feature maps are in turn analysed for features by further convolutional layers, to learn multiple higher-level assemblies of features, before then spatial downsampling to assist in broadening the spatial coverage of the feature learning while still capturing the entire information from the whole input sinogram. This learned analysis of feature hierarchies is analogous to perceiving real-world objects as first composed of atoms, then of higher-level groupings of atoms (molecules), then numerous various assemblies of molecules to form materials, all the way through to larger assemblies of materials that form larger scale objects. The convolutional encoding and downsampling continues up to a latent space composed of many feature maps of very limited spatial sampling, at which stage further processing layers are learned for that low level of spatial sampling. After latent space processing, a convolutional decoder is learned for image synthesis, with progressive increases in spatial sampling and corresponding decreases in the number of feature maps, to arrive ultimately at a single output high spatial resolution feature map, which is trained to be the final reconstructed image. This kind of architecture is referred to as a convolutional encoder decoder (CED).^41,42^

By analogy to conventional Fourier-based reconstruction methods, we can broadly regard a CED as learning from data how to perform an encoding transform to a latent space (by simple analogy, Fourier transforming to the frequency domain) and then decoding from the latent space to synthesise an image (by analogy, inverse Fourier transforming the processed frequency domain data).

The supervised training of a CED relies on an optimiser (such as the gradient-based Adam algorithm)^43^ to adapt the parameters of the encoder and decoder networks such that the output from a given input sinogram matches the associated target image for that sinogram, according to a mean square error (MSE) loss function. The target image used in supervised learning can be the ground-truth image (if known, *e.g.* by imaging known test objects, or by advanced Monte Carlo simulations)^44^ or a high quality image obtained by reconstructing high count data by other methods.

Strictly this CED network is not without a model—there are constraints on the range of possible output images that can be generated from this architecture, so the network still has what is known as an inductive prior or inductive bias. As can be seen in Figure 3, the output results from DeepPET can appear overly smooth, lacking in detail. Methods which followed, such as a modified implementation for long-axial FOV PET (also shown in Figure 3), and the earlier DPIR-Net,^39^ sought to improve DeepPET by modifying the loss function used during network training. Instead of just focusing on agreement between the network output and the label or target in a training set by MSE, these later methods also required agreement at a feature map level (perceptual loss, using a pretrained VGG^45^ network) as well as, for DPIR-Net, requiring the reconstruction outputs to be able to pass a discriminator test. The discriminator^46^ is a network which is trained to identify real high-quality images from ones generated by a network. This discriminator can hence be used to enhance the training of the generator (if a network’s output is classified as generated rather than real, this causes the optimiser to search for network parameters which generate outputs that look more real—to “fool” the discriminator). This is the well-known “generative adversarial network” (GAN) approach.^46^ Hence, the trained generator network can produce reconstructed images that look indiscernibly different to real high quality images.

The long-axial FOV modified implementation reports fast reconstruction times (~20× faster), relies on single slice rebinning (SSRB)^47^ to preprocess the fully 3D data into manageable 2D sinograms, and very conveniently enables scatter and normalisation corrections to be handled entirely by the learned CED network. It is however acknowledged, as visible in Figure 3, that the reconstruction accuracy of DeepPET and the modifications are not quite yet at the level to meet clinical requirements, but there is no reason why this should not be attained in future with improved training and dataset availability.

Table 1 summarises a number of the current direct AI methods. The notable aspects of these direct AI methods are that: (i) the majority only operate on 2D sinograms rather than fully 3D sets of sinogram data, (ii) many tens of thousands of training pairs are needed, (iii) training can be slow, (iv) reconstruction speed is typically fast. Perhaps one of the more notable methods is that of FastPET,^48^ as it makes direct use of image-space histoimages. Histoimages have the advantage of compressing time of flight (TOF) PET data into a 3D image-space format. By effectively compressing large TOF datasets into 3D images, relatively straightforward image-to-image deep learning mappings (such as U-Nets)^49^ can be used for fast 3D image reconstruction. This method has been successfully extended and further improved,^50,51^ demonstrating good potential.

**Table 1.** Example direct AI reconstruction methods

Name	Architecture [total parameters]	Input	Target	Loss function and optimiser (epochs) validation	Number of samples (training, validation, test)
AUTOMAP Zhu et al. 2018^10^	Two fully connected layers and CNN [∼800M]	2D noisy sinograms	T1w brain images (128 × 128)	MSE with L1-norm penalty on network weights in final hidden layer RMSProp (100) Validation not used	50,000 n/a 1
DeepPET Häggström et al. 2019^11^	CED [>60M]	2D noisy sinograms (269 × 288)	Ground-truth PET images (128 × 128)	MSE Adam (150) Validation used	203,305 (70%) 43,499 (15%) 44,256 (15%)
DPIR-Net Hu et al. 2020^39^	CED [>60M] Discriminator [>3.5M]	2D noisy sinograms (269 × 288)	Ground-truth PET images (128 × 128)	Wasserstein GAN + VGG + MSE Adam (100) Validation not used	37,872 (80%) n/a 9468 (20%)
CED extended to SSRB sinograms from large FOV PET Ma et al. 2022^40^	CED [∼64M]	2D noisy sinogram (269 × 288)	Reconstructed PET images using OSEM + PSF TOF from list-mode data (128 × 128)	MSE + SSIM+ VGG Adam (300) Validation used	35,940 (76%) 5590 (12%) 5590 (12%)
FastPET Whiteley et al. 2021^48^	U-Net [∼20M]	Noisy histo-image slices + attenuation map slices (2 × 440 × 440 × 96)	Image slices reconstructed using OSEM + PSF (440 × 440 × 96)	MAE + MS-SSIM Adam (500) Validation used	20,297 slices (74%) 1767 slices (6%) 5208 slices (20%)

CNN refers to a convolutional neural network, CED refers to a convolutional encoder-decoder. VGG in this table refers to perceptual loss based on a VGG network.

3D, three-dimensional; FBP, filtered backprojection; MLEM, maximum likelihood–expectation maximisation; OSEM, ordered subsets expectation maximisation; PSF, point spread function.

### Iterative AI methods for PET reconstruction

Just as conventional reconstruction algorithms can be broadly categorised as either direct (a non-iterative sequence of programming operations) or iterative (a sequence of coding steps repeated a variable or indefinite number of times), so also there are comparable categories in deep learning. This section now covers the iterative, or unrolled, AI methods.

**Figure 4.**
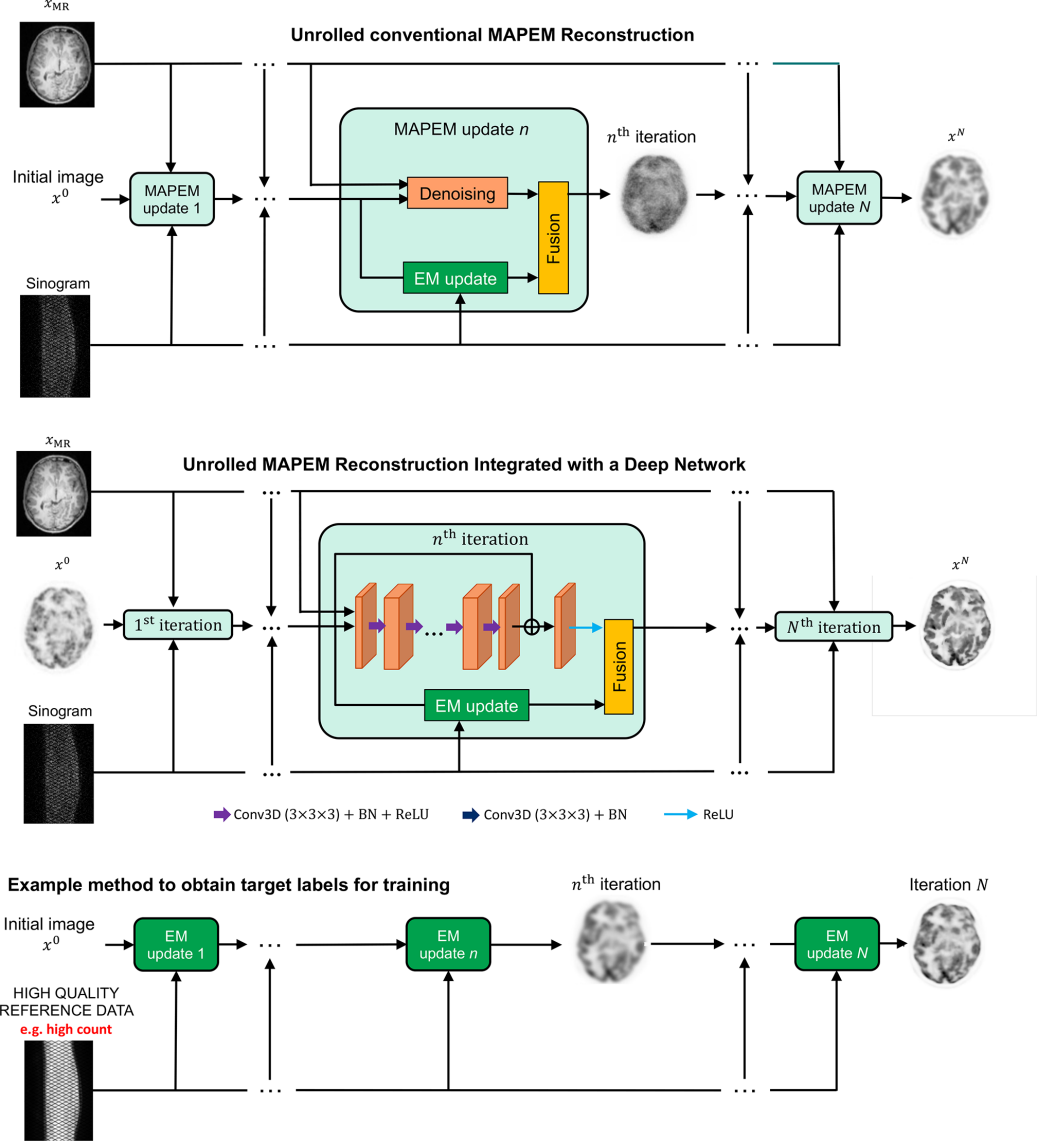
The example of FBSEM-Net. Row 1 shows an unrolled iterative MAPEM reconstruction, row 2 shows integration of a deep network (FBSEM-Net) and row 3 shows an example way of generating reference target labels for the network. Here, the EM update improves agreement of the image with the measured sinogram data, and can be called a data-fidelity based update. In an MRI reconstruction context, a data-fidelity update is usually achieved by a least-squares gradient descent step. EM, expectation maximisation; MAPEM, maximum a posteriori expectation maximisation.

**Figure 5.**
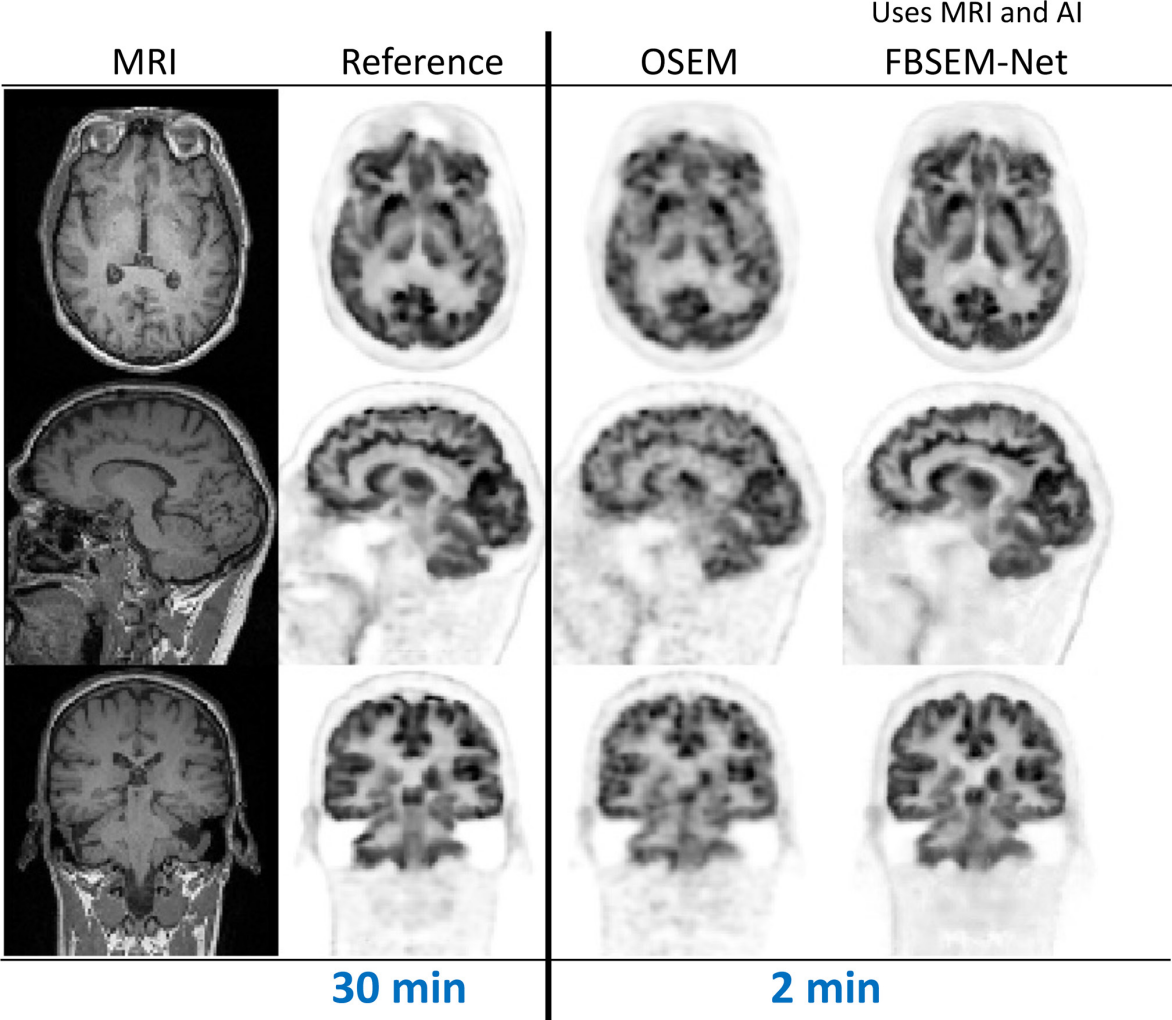
Example results from FBSEM-Net.^52,53^ FBSEM-Net offers improved image quality when reconstructing data equivalent to just 2 min of scanning time, with image quality competitive to the reference reconstruction from ~15 times more data (30 min of data). The results shown are for “test time” reconstructions (*i.e.* for reconstruction of data not included in the training of FBSEM-Net). OSEM, ordered subsets expectation maximisation; FBSEM, forward backward splitting expectation maximisation.

Figure 4 shows how a conventional iterative MAPEM algorithm^35^ can be unrolled into a sequence of modules or blocks (from left to right in the figure, one for each update of the image). Each module performs refinement of the current image estimate to improve its agreement with the data (*e.g.* based on the EM update derived for Poisson distributed data) whilst also balancing this with agreement with the prior via an analytically derived denoiser (*e.g.* derived from a quadratic penalty, which encourages images to have small local differences between pixel values). Hence, these conventional iterative methods are simply a sequence of steps, layer after layer of processing, and can be regarded as fixed, non-trainable, deep networks. The second row of Figure 4 shows how the denoising (regularisation) step in MAPEM can be replaced with a trainable deep network, to learn from paired data examples how to relate noisy image updates to their high-quality counterparts. The third row of the figure shows how reference high quality reconstructions (*e.g.* from higher count sinogram data) can be used to provide training targets for the unrolled network. Figure 5 shows the kind of results obtained from training an unrolled iterative method (MAPEM) with a learned regulariser, based on 45 3D training examples.

**Figure 6.**
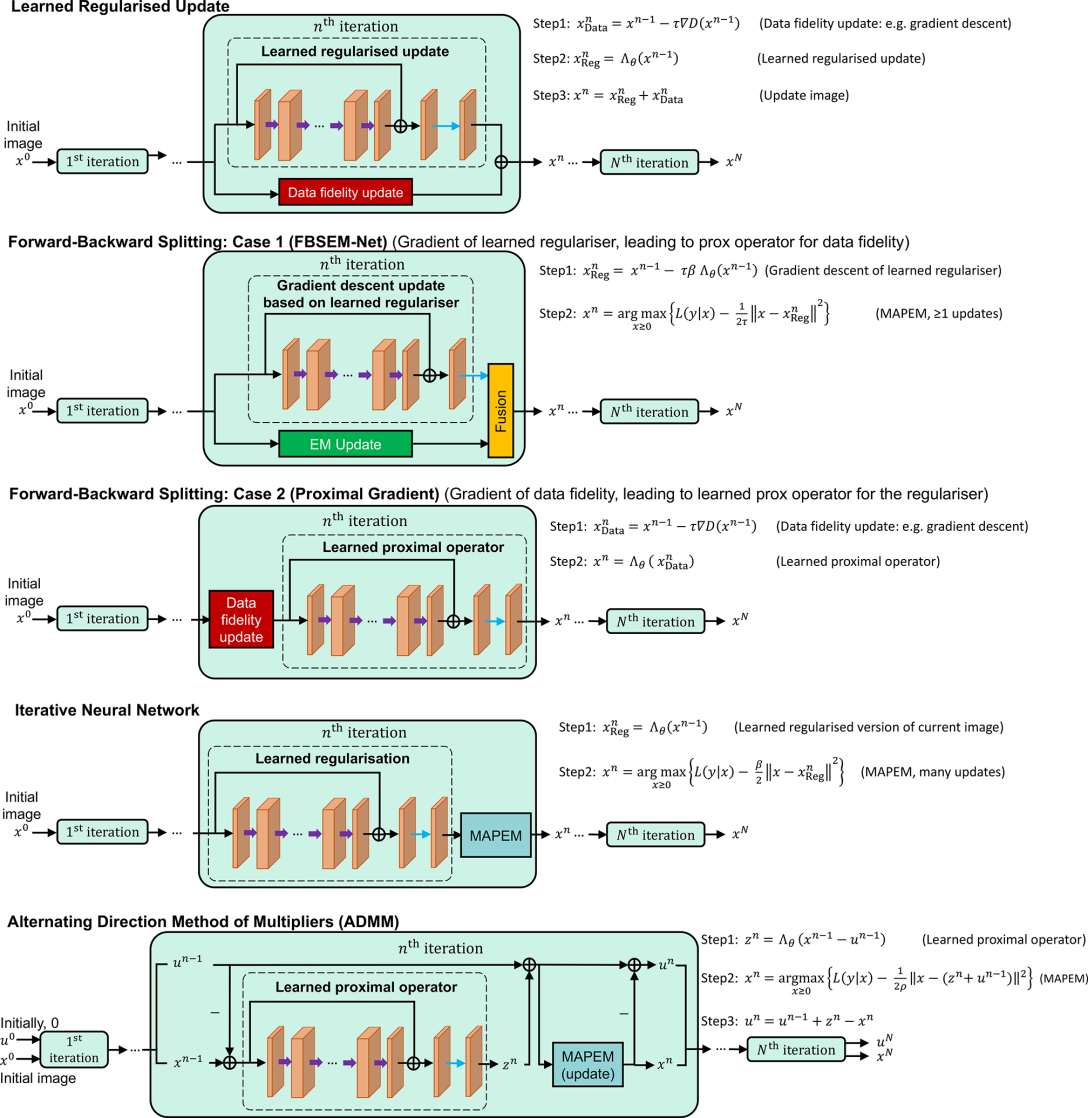
Five example unrolled iterative image reconstruction methods with deep networks embedded. Row 1 corresponds to taking a current image and updating it according to the data (a gradient descent step to increase agreement with the measured data), and also updating it according to the prior knowledge of how the image should appear (simply using a deep network to provide this increased agreement). Row 2 is very similar, but uses an analytically derived way for combining the two updates (rather than a simple addition, as in row 1). Row 3 corresponds to a deep learned version of a proximal gradient update. The example in row 4 is the sequential method but now using an analytical form of the update. Finally, row 5 shows how the ADMM method can have a network embedded, and it amounts to the same approach as in row 4, but with use of a residual image, *u*.

A range of unrolled reconstruction methods have now been developed, and some key methodologies are shown in Figure 6. Each one performs regularisation via a learned network in different positions in the processing pipeline. Examples include gradient descent with a learned regulariser (comparable to EM-Net^54^ for PET, or similar to a variational neural network (VNN) for MRI),^55^ learning the gradient of the regulariser (*e.g.* FBSEM-Net),^52^ learning the proximal operator and learning to denoise (Iterative Neural Network).^56^ A further example would be an unrolled alternating direction method of multipliers approach, with a deep network embedded (such as deep ADMM-Net).^57^

**Figure 7.**
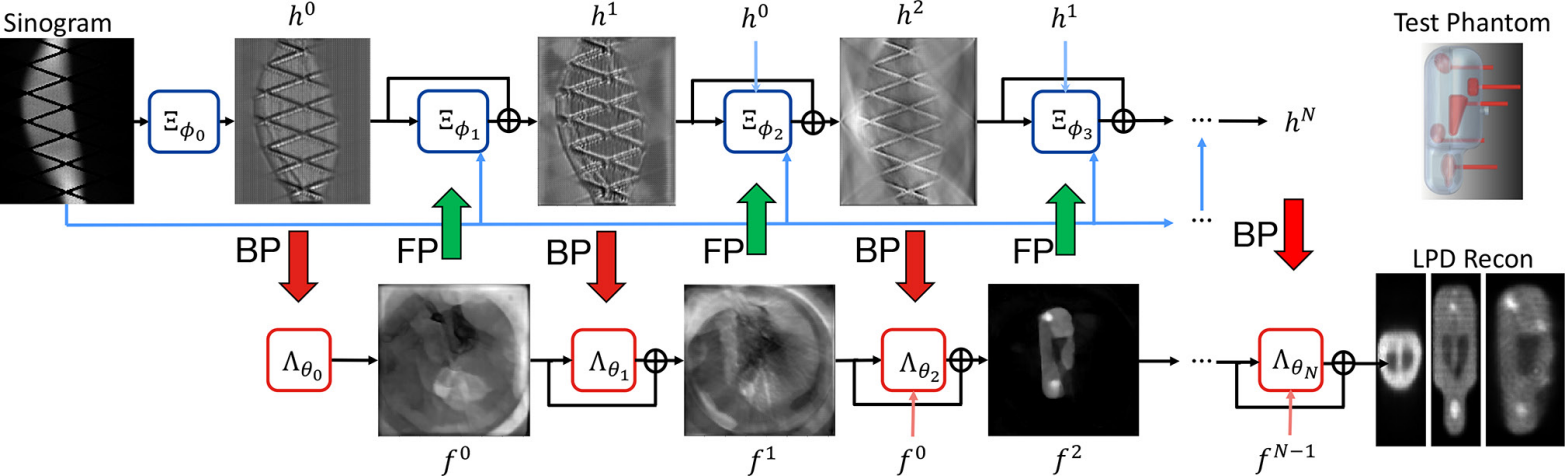
LPD^58^ when applied to example PET phantom data.^59^ Figure adapted from data courtesy Massimiliano Colarieti-Tosti. The overall processing pipeline effectively has two rails: the top rail conducts sequential processing of the sinogram data and the lower rail conducts sequential processing of the image. These two processing subpipelines are interconnected by forward and backprojection operators (FP and BP) to map between the image domain and data domain. BP, backprojection; FP, forward projection; LPD, learned primal dual; PET, positron emission tomography.

One final example of unrolled methods is the learned primal dual (LPD) method,^58,59^ based on the primal dual hybrid gradient (PDHG) method,^60^ but with learning of the processing in both the primal (image) and dual (sinogram) domains, as show in Figure 7. Table 2 summarises a variety of unrolled, iterative, deep learning reconstruction methods for PET, where in one case a transformer architecture is used. Whereas CNNs often use small kernels in each network layer (hence needing many layers to encode an input to a latent representation), transformers use long-range comparisons of features across the entirety of an image in a single layer, via their attention mechanism. Transformers are broadly analogous to a trainable version of state-of-the-art conventional image processing via non-local means, where an image patch is compared with all other patches in an entire image for the purpose of contextualisation / denoising. Table 3 conveys the advantages of unrolled methods in general compared to direct AI methods: use of the physics model, less training data needed, and practical for fully 3D image reconstruction. Nonetheless, it is important to note that not only are these unrolled methods computationally intensive to train, even at run time (test time on new data) they can potentially be slow to run, being dependent on multiple iterations of forward and backprojections. It is though worth noting that differing training methods are available, some of which are more practical and less memory intensive.^63,64^

**Table 2.** Unrolled methods for AI in PET image reconstruction.

Name	Architecture [total parameters]	Input	Target	Loss function and optimiser (epochs) validation training method	Number of samples (training, validation, test)
EM-Net Gong et al. 2019^54^	10 modules U-Net shared [∼2M]	Previous output/iteration	3D high count reconstruction (128 × 128 × 46)	MSE Adam *Validation not used* Gradient truncation	16 n/a 1
MAPEM-Net Gong et al. 2019^61^	8 modules U-Net not shared [∼16M]	Current output from a block	3D high count reconstruction (128 × 128 × 105)	MSE [Details not specified] *Validation not used* End-to-end	18 n/a 1
FBSEM-Net Mehranian and Reader 2020^52^	10 modules CNN shared [∼77k]	Previous output / iteration	3D high count reconstruction— cropped (114 × 114 × 128)	MSE Adam (200) *Validation used* Gradient truncation	45 5 5
Iterative Neural Network Lim et al. 2020^56^	10 modules CNN not-shared [∼40k]	Current output from a block	3D true activity image (200 × 200 × 112)	MSE Adam (500) *Validation not used* Sequential training	4 n/a 1
TransEM Hu and Liu 2022^62^	10 modules Swin Transformer [details not specified]	Previous output / iteration	2D high count reconstruction	MSE Adam Validation used Gradient truncation	510 30 60

AI, artificial intelligence; CNN, convolutional neural network; 2D, two-dimensional; 3D, three-dimensional; PET, positron emission tomography.

**Table 3.** Broad level comparison of core approaches to using AI in PET image reconstruction.

	Direct AI methods	Unrolled AI methods	AI methods without training data
Interpretability	Low	Good	Good
Use of physics model	No	Yes	Yes
Generalisation (outside training distribution)	Can be challenging	Better than direct methods	Good
Suitability for fully 3D image reconstruction	Challenging	Yes	Yes
Typical number of training pairs	>10,000 (2D reconstruction)	~50 (3D reconstruction)	1
Inference time/time to reconstruct from new data	Fast	Potentially the slowest	Faster than unrolled methods, depending on implementation
Expected image quality if within training distribution	Potentially the best	Good	Does not benefit from population data

2D, two-dimensional; 3D, three-dimensional; AI, artificial intelligence; PET, positron emission tomography.

**Figure 8.**
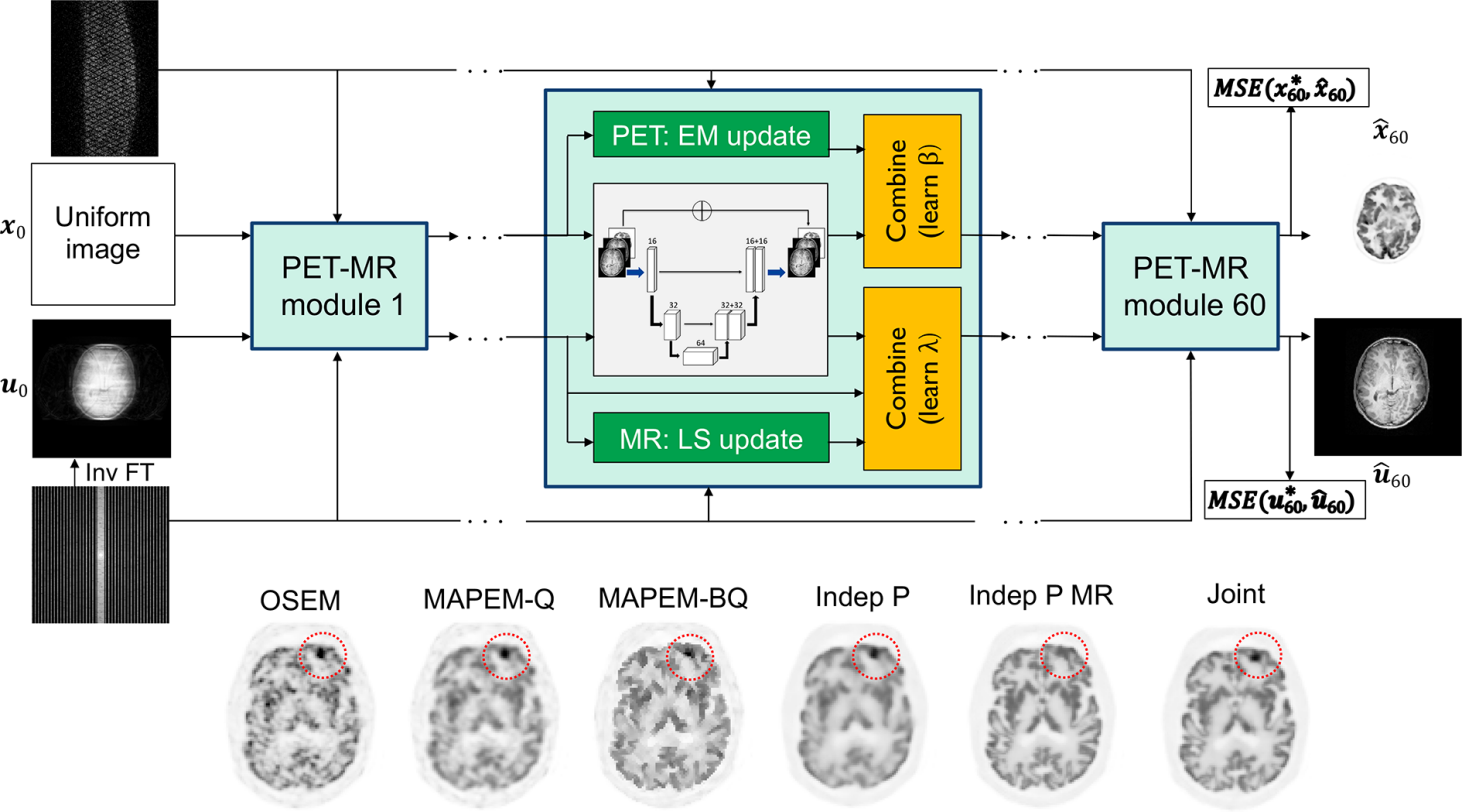
The example of unrolled joint PET-MR reconstruction from Corda-D’Incan et al.^65^ Example results are shown, comparing OSEM with MAPEM (Q, using a quadratic penalty, BQ using a Bowsher MRI-guided quadratic penalty), and with independent deep learned unrolled reconstruction from PET data only (**P**) and with MR guidance (P MR). The final result shown is the joint reconstruction of the PET data—revealing preservation of a PET-unique lesion (absent in the MR) whilst retaining good image quality globally—effectively seeking the best aspects of “Indep P” and “Indep P MR” simultaneously. OSEM, ordered subsets expectation maximisation; MAPEM, maximum a posteriori expectation maximisation; MSE, mean square error; PET, positron emission tomography.

We finish this section by mentioning some of the more advanced unrolled iterative methods, which seek to reconstruct PET and MR data simultaneously, as shown in Figure 8. This joint PET-MR unrolled iterative reconstruction method^65^ learns a joint PET-MR regularisation, seeking to benefit from the common information present in both the PET and MR data, whilst preserving the crucial differences between PET and MR images.

## AI methods without training data

We have so far covered direct AI methods and iterative AI methods (which integrate deep learning into otherwise standard iterative image reconstruction schemes). Both categories rely on training data for supervised learning of the parameterised deep networks used in each type of method. A third major category of research into AI for PET image reconstruction is the case where no training data are needed at all, but instead, deep learning methodology is used either to learn a representation of the image (for just the measured dataset in hand), or to learn a dedicated reconstruction operator for the data in hand to reconstruct. Hence, no external training data are needed, and AI is exploited instead to solve processing problems. As there are no example or external training data, such methods do still need a model of the imaging physics and specification of an image reconstruction objective (*e.g.* ML, or MAP).

### AI for image representations

The deep image prior^66^ (DIP) was proposed in 2017–18, and delivered surprising results for image denoising, inpainting and super resolution in the absence of training data. The method exploits the inductive prior of a CNN, whereby the sequence of convolutional layers with non-linearities can generate, with appropriate initialisation, a range of image outputs that are regularised in the sense that they readily locate within broadly feasible manifolds occupied by medical or natural images. Typically, the noisy data are used as the target (or, for PET reconstruction, the noisy sinogram is used as the target after first applying the forward model to the generated output reconstruction). The regularisation benefit depends entirely on the level of training—if the networks are trained towards convergence, then there will be near perfect matching of the noisy data. This still solves the inverse problem (*e.g.* for PET image reconstruction),^67^ but would eliminate any regularisation benefit afforded by a CNN representation of the image.

**Figure 9.**
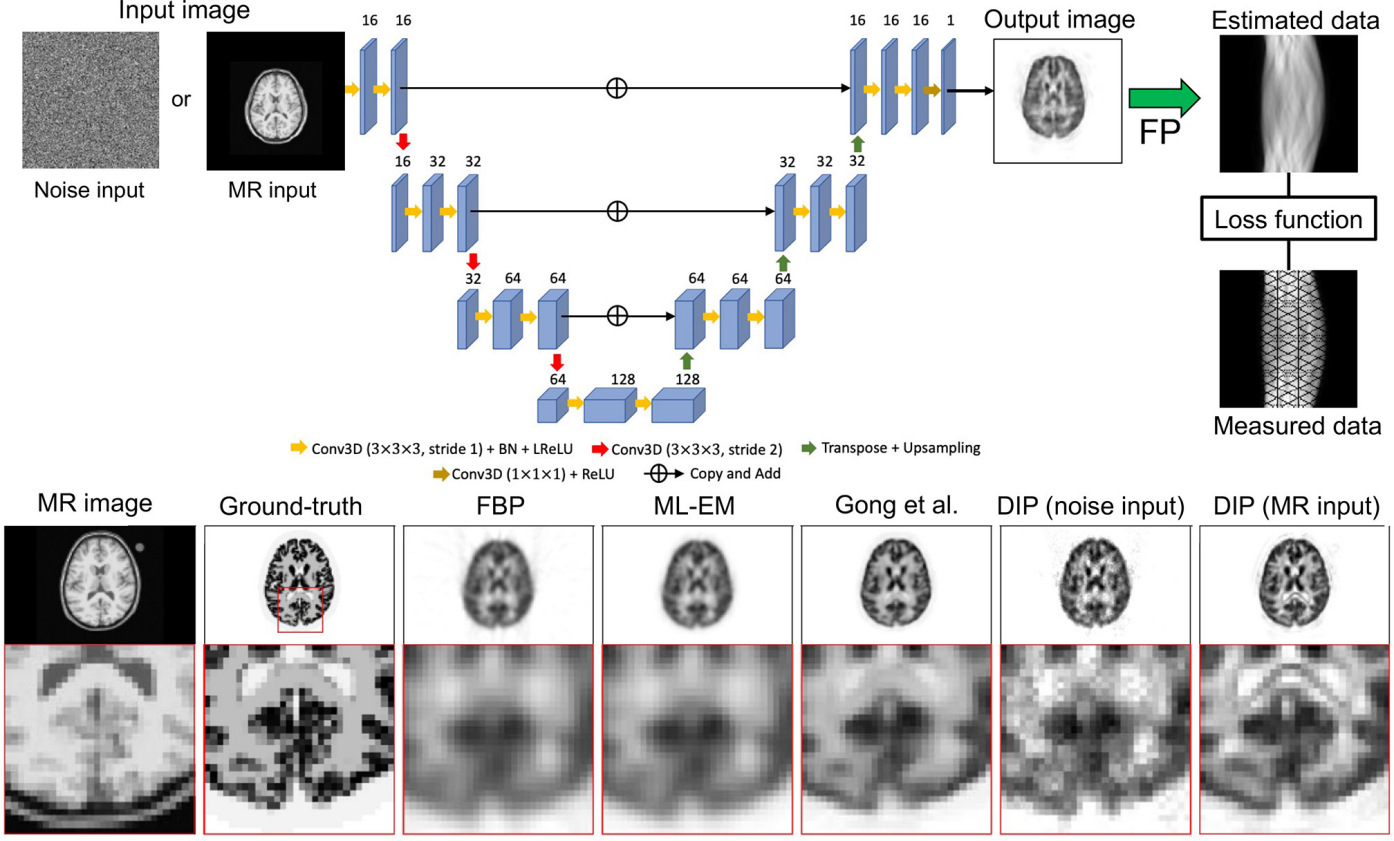
Basic use of the DIP for PET reconstruction, from Hashimoto et al.^68^ FBP, filtered backprojection; DIP, deep image prior; FP, forward projection; PET, positron emission tomography; MLEM, maximum likelihood - expectation maximisation.

Figure 9 shows a simple example of using the DIP for PET image reconstruction.^68^ Either random noise or the patient’s MR image are fed into a randomly initialised CNN, such as a U-Net.^49^ The output image is then forward modelled, and the training loss function for the CNN is the MSE between the forward modelled data and the actual measured data. Hence, with increasing optimisation of the CNN’s parameters, the network will produce an image which, when forward projected, agrees (to within the range of the forward operator) with the measured data. Early termination is therefore needed to see any regularised result. If run to convergence, the CNN representation is no different to a pixel or voxel grid representation, and the reconstruction algorithm amounts to using a deep learning optimiser (such as Adam)^43^ to find a least-squares (or for Poisson data, a maximum Poisson likelihood) estimate. Prior even to the work of Hashimoto et al,^68^ Gong had proposed a more elaborate optimisation framework using a CNN representation of the image.^69^ This method was designed to avoid the need for embedding the system model into the deep network and hence avoid backpropagation of loss function gradients through the system model during training.

In seeking to resolve the issue of needing to stop the DIP training early, Bayesian methodology has been investigated by many. Examples include using a prior for the network parameters (and even using posterior distributions to quantify and make use of the uncertainty^70,71^) and use of stochastic gradient Langevin dynamics (SGLD) for fully Bayesian posterior inference via the DIP.^72,73^ A further approach has been Stein’s unbiased risk estimator (SURE) as the loss function for training the DIP (DIP-SURE) instead of using an MSE loss, again to avoid the overfitting problem.^74^

**Figure 10.**
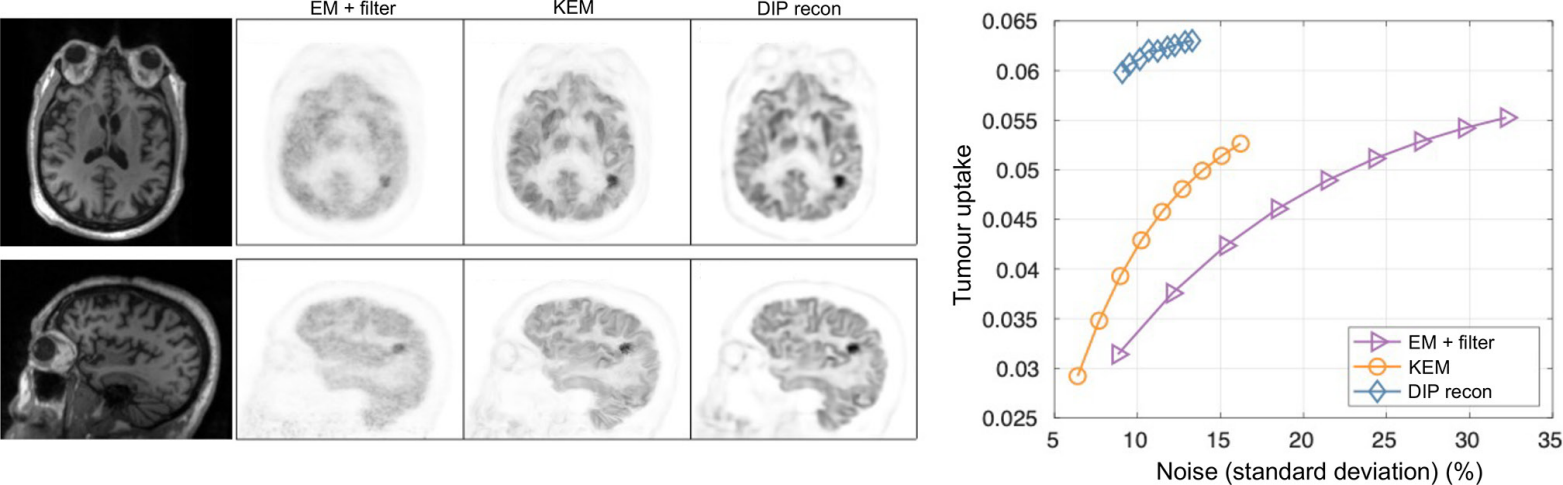
Results of using the DIP approach with the kernel method for direct Patlak reconstruction (“DIP recon”), from Gong et al.^75^ DIP, deep image prior; EM, expectation maximisation; KEM, kernel method.

More recently, Gong et al have taken the DIP method further, by combining it with the kernel method, and then also with direct kinetic parametric estimation.^75^ Figure 10 shows the impressive results achieved for direct estimation of Patlak slope ( $K_{i}$ ) images.

Table 4 summarises the methods that exploit the DIP for PET image reconstruction, including also some AI-assisted variations of KEM, which will be covered later.

**Table 4.** DIP and AI-assisted KEM methods for PET image reconstruction.

Name	Architecture [total parameters]	Input	Target	Loss function and optimiser (epochs)
Deep Image Prior Gong et al. 2019^69^	U-Net [∼2M]	3D anatomical prior (MRI)	An EM iteration in 3D (192 × 192 × 128)	MSE L-BFGS (20 epochs per ADMM iteration, 100 ADMM iterations in total)
Deep Image Prior – with non-negative matrix factorisation Yokota et al. 2019^76^	3 U-Nets combined in parallel [∼4M]	Noise	Spatial factors— homogeneous tissues with kinetic parameters (128 × 128 × 3)	MSE Adam (20,000)
Deep Image Prior, kernel layer – direct kinetic parametric estimation Gong et al. 2022^75^	U-Net [∼2M]	T1w 3D MR image	A (dynamic) EM iteration in 3D (128 × 128 × 96 × 7)	MSE L-BFGS (20 epochs per ADMM iteration, 100 ADMM iterations in total)
Neural KEM Li et al. 2022^77^	U-Net [∼2M]	1-hour composite frames 3D PET images (192 × 192 × 47 × 97)	An EM iteration of coefficient image update	An EM-type surrogate function Adam
Deep Kernel Representation Li et al. 2022^78^	U-Net [∼2M]	Low-count 3D dynamic PET images	An EM iteration of coefficient images update	MSE [Details not specified]
Deep Image Prior with forward projection Hashimoto et al. 2022^68^	U-Net [∼2M]	Noise or T1w MR image	2D noisy PET sinogram series (256 × 256 × 64)	L2 loss L-BFGS
Uncertainty-Informed Bayesian Deep Image Prior Sudarshan et al 2022^71^	U-Net [details not specified]	3D MR image	An EM iteration in 3D	MSE loss with variance term (BDIP loss) in the first iteration, and BDIP loss + uncertainty-weighted MSE loss in the later iterations Adam
Deep Image Prior with DeepRED Shen et al. 2023^73^	Generator: U-Net [∼19M] Denoiser: U-Net [∼1M]	Apply transpose of the system matrix to the 2D noisy sinogram (backprojection)	2D whole-body PET image slices at 10M count level (192 × 192)	MSE (sinogram domain) + RED loss (image domain) Adam

2D, two-dimensional; 3D, three-dimensional; AI, artificial intelligence; DIP, deep image prior; BDIP, Bayesian deep image prior; EM, expectation maximisation; KEM, kernel method, PET, positron emission tomography.

### AI for reconstruction operators

**Figure 11.**
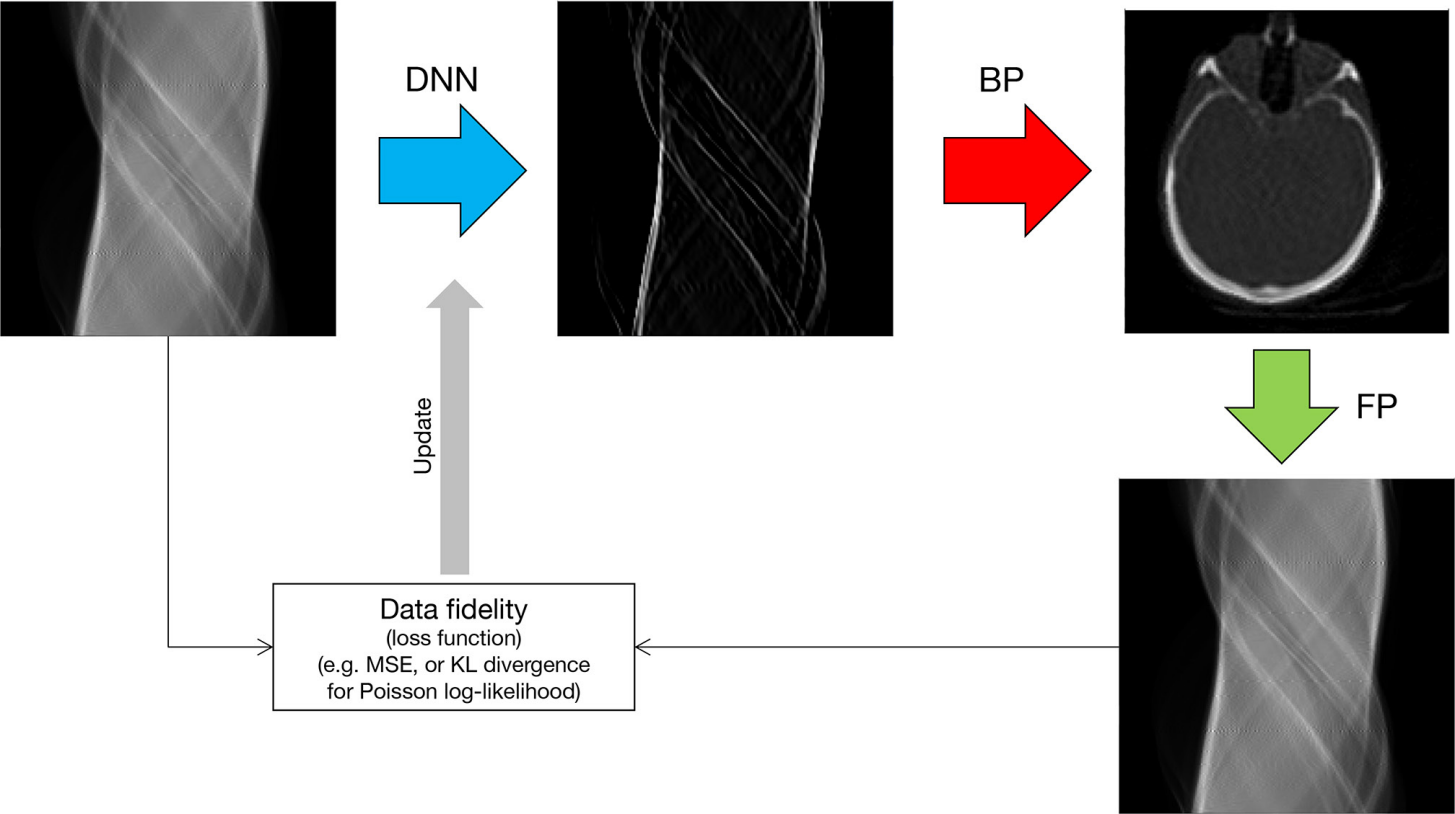
Illustration of learning a deep reconstruction operator (a DNN) to operate on sinogram data such that, when backprojected, an image is obtained.^79^ This is an example of self-supervised deep-learned FBP, from Reader,^79^ show just by way of example for the case of a simulated CT slice, but the method is generally applicable. DNN, deep neural network; BP, backprojection; FP, forward projection.

An alternative use of AI in the absence of training data is to define a deep network to perform processing at certain stages in the image reconstruction pipeline. Figure 11 gives the example of deep-learned FBP (DL-FBP): a deep network is used to process a sinogram in such a way that, when the processed sinogram is backprojected, a reconstructed image is obtained. The image is a reconstruction, as the training of the deep network is based on minimising the discrepancy (loss) between the forward projection of the image and the actual input measured data. This is an example of filling in a potential gap in our knowledge by deep learning: we know what we require (a reconstructed image that agrees with the sinogram data according to some reconstruction objective function), but we may not know how to do the processing of the data to achieve this. AI can be used to solve such tasks, tapping into the optimisation methods that have been so successful in deep learning. An important further development on this theme is to combine an image-space deep network with a data-space network,^79^ to obtain DL-FBP-F. This approach, just as DL-FBP, can be implemented just by self-supervised learning from the dataset in hand, or optionally combined with supervised learning. If used exclusively with supervised learning, then the DL-FBP-F method becomes similar to just one module of an unrolled method, but with much reduced computation compared to the many modules usually employed in unrolled methods, which may reduce their level of adoption.

### AI-assisted KEM

**Figure 12.**
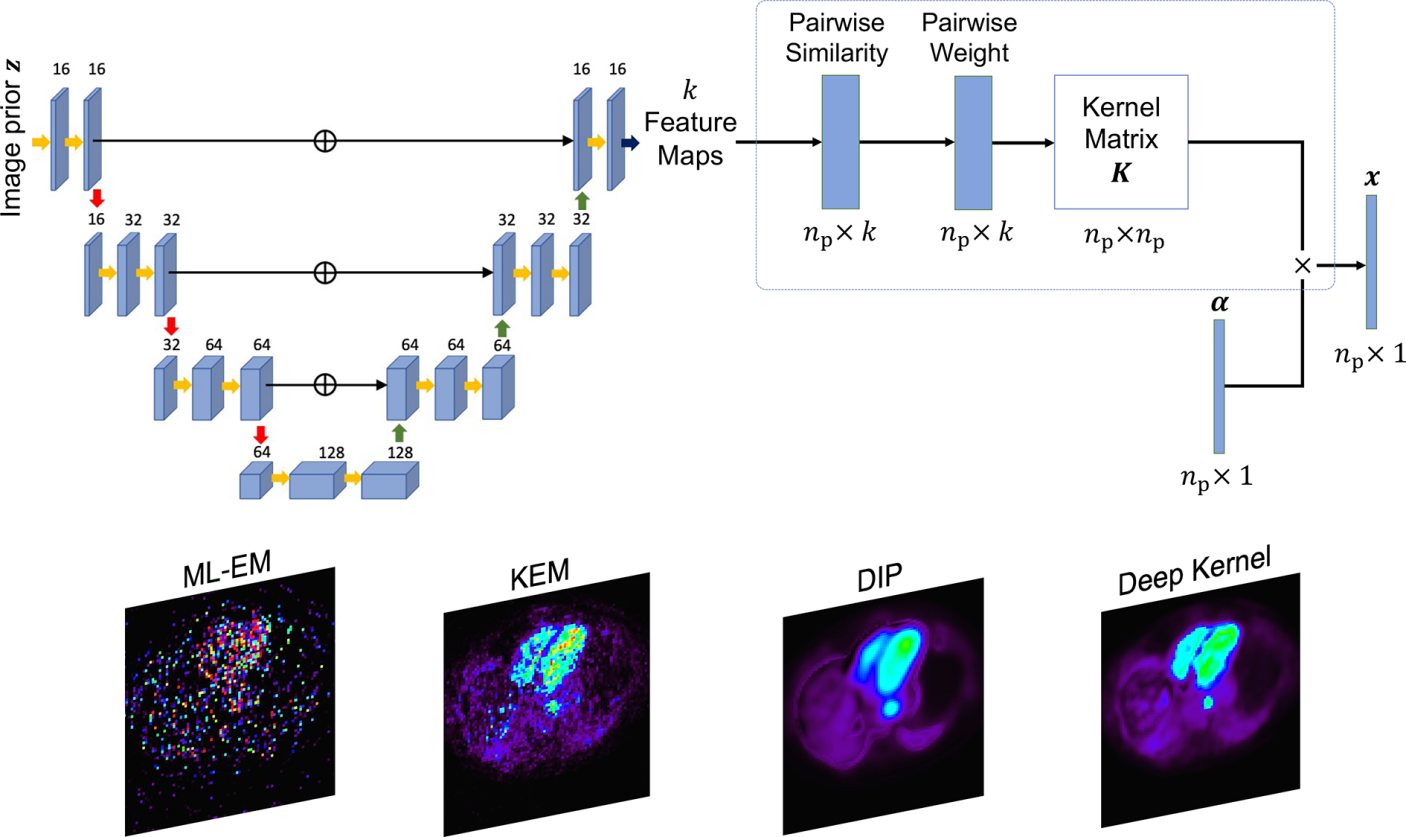
Deep kernel representation by Li and Wang.^78^ A U-Net operates on a prior image (*e.g.* a 3-frame dynamic image series) to output a set of k different feature maps (a feature vector with k elements for each of the $n_{p}$ pixels). The similarity of feature vectors between pixels is then used to construct the kernel matrix K , subsequently used in standard KEM. Standard KEM estimates a coefficient vector α , such that the reconstructed image x is found by Kx . The crucial training of the U-Net is based on mapping a 10x count-reduced reconstruction to a full-count reconstruction of the unique data being reconstructed. DIP, deep image prior; KEM, kernel method; MLEM, maximum likelihood–expectation maximisation.

A final important category, related to the example of using the kernel method with the DIP as previously shown in Figure 10 (and Table 4), is using a deep network to learn the best features to use for forming the kernel matrix used in KEM, called the deep kernel representation.^78^ Training data are derived from the single dataset to reconstruct (by generating low-count versions of the data), and the method elegantly learns what are the most appropriate features to pick out from an input prior image such that when these features are used in a voxel-level similarity comparison to form spatial basis functions for KEM, the output image best matches the full-count high quality reference. These basis functions are then used with standard KEM for reconstructing the whole dataset. Example results from this deep kernel representation are shown in Figure 12, where it outperforms the conventional DIP (with early termination and oversmoothness) as well as conventional KEM based on 3-point TAC feature vectors.

## Opportunities, challenges and summary

These significant advances in using AI for PET image reconstruction are not without concerns. A well-known concern, *e.g.* demonstrated for AI-based MR reconstruction, is the possibility of hallucinations (false positives) and the possibility of deletions (false negatives) from images.^80^ Whereas these effects were always possible with conventional reconstruction (a noise spike looking like a lesion, or noise and smoothing seeming to remove a lesion), in conventional reconstruction it was always easy for an observer to assess the quality of an image, and hence make allowances for such occurrences. With the improved image quality from AI methods, hallucinations or deletions may not be at all appreciable based on the overall impression of image quality. A potential solution would be to take the Bayesian approaches previously described (developed also in the MR context)^81^ and generate additional images reflecting the level of uncertainty in the reconstructed image values. Yet that would increase the image reading burden for radiologists. Nonetheless, Bayesian deep learning approaches, or methods based on deep ensembles^82^ or even drop-out,^83^ are ways to reveal the epistemic uncertainty in reconstructed images. Related to this is a very recent advance in generative AI modelling: diffusion models (popularised by image generation models such as Stable Diffusion).^84^ These models can be trained to provide prior probabilities for high quality images, and as such are now finding their way into image reconstruction^85,86^ and producing state-of-the-art results (as so far demonstrated for MRI reconstruction), providing also uncertainty estimates.^81^ Early work in PET has already begun in a post-processing context.^87^

For PET image reconstruction, there also remains the need for widely accepted benchmark datasets and tests (as increasingly used in MRI),^88,89^ needed not just for conventional methods but also AI approaches. Early developments in high resolution PET phantoms^90^ will need much further development, alongside pooling of large real datasets.

A further area of development will be in full exploitation of all types of available data, so that not only supervised learning can be carried out, but also self-supervised learning.^79^ This would allow larger datasets to be used in the absence of high-quality references. Instead of providing high-cost example paired inputs and outputs, only example input data are provided, but crucially with instructions on how to create paired targets automatically for the inputs, hence creating data for supervised learning. This can be for a pretext task, or indeed directly for a useful end-point task of image reconstruction (as previously shown in Figure 11). Self-supervised approaches with pretext tasks have enabled pretraining of large language models, including powerful pretrained transformer-based generative architectures^91^ such as GPT-3^92^ and GPT-4,^93^ which have now become widely known with significant impact.

One other aspect to note is how AI opens new performance possibilities for PET processing and reconstruction in general. To give just one example area, training data can be used to deliver improved performance for separation of multiplexed (*e.g.* dual tracer^94,95^) PET image data.^96,97^

In summary, AI now plays a key role in PET image reconstruction. It has introduced the possibility of making use of high-quality reference data to inform and improve the image reconstruction algorithms that are presently in use. Or, if data external to the patient are unavailable or of concern to use, AI can also provide many methods that only use a patient’s own data, potentially alleviating concerns of robustness.
